# Arbutin: Occurrence in Plants, and Its Potential as an Anticancer Agent

**DOI:** 10.3390/molecules27248786

**Published:** 2022-12-11

**Authors:** Lutfun Nahar, Afaf Al-Groshi, Anil Kumar, Satyajit D. Sarker

**Affiliations:** 1 Laboratory of Growth Regulators, Palacký University and Institute of Experimental Botany, The Czech Academy of Sciences, Šlechtitelů 27, 78371 Olomouc, Czech Republic; 2 Centre for Natural Products Discovery, School of Pharmacy and Biomolecular Sciences, Liverpool John Moores University, James Parsons Building, Byrom Street, Liverpool L3 3AF, UK; 3 Faculty of Pharmacy, Tripoli University, Tripoli 42300, Libya; 4 Department of Biotechnology, Government V. Y. T. PG Autonomous College, Durg 491001, Chhattisgarh, India

**Keywords:** arbutin, anticancer, distribution, hydroquinone

## Abstract

Arbutin, a hydroquinone glucoside, has been detected in ca. 50 plant families, especially in the plants of the Asteraceae, Ericaceae, Proteaceae and Rosaceae families. It is one of the most widely used natural skin-whitening agents. In addition to its skin whitening property, arbutin possesses other therapeutically relevant biological properties, e.g., antioxidant, antimicrobial and anti-inflammatory, as well as anticancer potential. This review presents, for the first time, a comprehensive overview of the distribution of arbutin in the plant kingdom and critically appraises its therapeutic potential as an anticancer agent based on the literature published until the end of August 2022, accessed via several databases, e.g., Web of Science, Science Direct, Dictionary of Natural Products, PubMed and Google Scholar. The keywords used in the search were arbutin, cancer, anticancer, distribution and hydroquinone. Published outputs suggest that arbutin has potential anticancer properties against bladder, bone, brain, breast, cervix, colon, liver, prostate and skin cancers and a low level of acute or chronic toxicity.

## 1. Introduction

**Figure 1:**
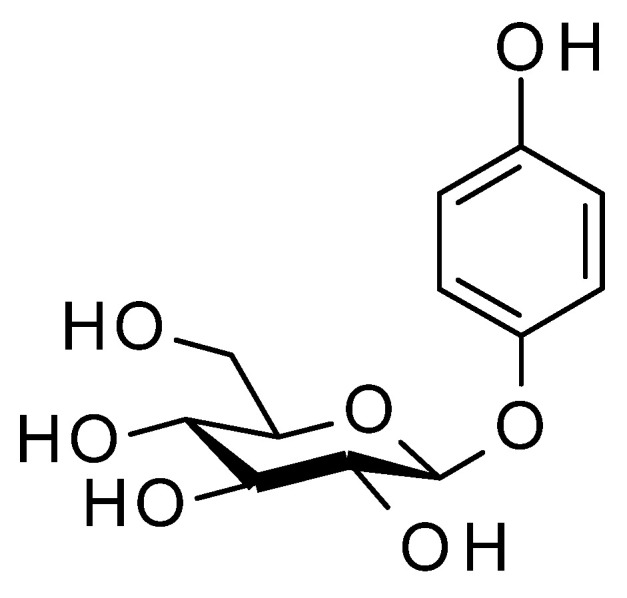
Arbutin (**1**).

Arbutin (**1**, C_12_H_16_O_7_), also known as β-arbutin, is a hydroquinone glucoside (Figure 1). This compound was first reported from the leaves of *Arbutus unedo* L. (family: Ericaceae) [1]. Arbutin structurally differs from its isomer α-arbutin by the presence of a β-glucose unit instead of an α-glucose one. Since its discovery, arbutin (**1**) has been detected in ca. 50 other plant families. As this glycoside (**1**) is capable of inhibiting melanin production by inhibiting tyrosinase, it has long been used as a skin whitening (depigmenting) agent in various commercially available topical cosmetic products [2,3]. It should be mentioned here that tyrosinase is a multi-copper enzyme that plays a pivotal role in melanogenesis and enzymatic browning. The objectives of this review are to extensively explore, for the first time, the distribution of arbutin (**1**) in the plant kingdom (Table 1) and critically appraise its therapeutic potential as an anticancer agent. In order to achieve these objectives, an extensive literature search was conducted covering the literature published until the end of August 2022, accessed through several databases, e.g., Web of Science, Science Direct, Dictionary of Natural Products, PubMed and Google Scholar, and using the keywords, arbutin, cancer, anticancer, distribution and hydroquinone.

## 2. Distribution of Arbutin (1) in the Plant Kingdom

Arbutin (**1**) is widely distributed in the plant kingdom (Table 1) [4,5,6,7,8,9,10,11,12,13,14,15,16,17,18,19,20,21,22,23,24,25,26,27,28,29,30,31,32,33,34,35,36,37,38,39,40,41,42,43,44,45,46,47,48,49,50,51,52,53,54,55,56,57,58,59,60,61,62,63,64,65,66,67,68,69,70,71,72,73,74,75,76,77,78,79,80,81,82,83,84,85,86,87,88,89,90,91,92,93,94,95,96,97,98,99,100,101,102,103,104,105,106,107,108,109,110,111,112,113,114,115,116,117,118,119,120,121,122,123,124,125,126,127,128,129,130,131,132,133,134,135,136,137,138,139,140,141,142,143,144,145,146,147,148,149,150,151,152,153,154,155,156,157,158,159,160,161,162]. While the plants from the families, Asteraceae, Ericaceae, Proteaceae and Rosaceae are the main sources, to date, at least 45 other plant families have been reported to produce this glycoside (Table 1). In the Asteraceae, the genera *Ainsliaea* [7], *Artemisia* [20], *Centaurea* [46], *Mutisia* [80], *Petasites* [92], *Podospermum* [98] and *Serratula* [136] are known to produce arbutin (**1**), while the genera *Arbutus* [12], *Arctostaphylos* [15], *Arctous* [19], *Calluna* [17], *Pyrola* [101], *Rhododendron* [125] and *Vaccinium* [147] from the family Ericaceae are seven other major sources thereof (Table 1). Plants from at least seven genera within the Proteaceae, e.g., *Bellendena* [25], *Cenarrhenes* [25], *Grevillea* [59], *Hakea* [60], *Heliciopsis* [63], *Leucadendron* [73] and *Persoonia* [25] biosynthesize arbutin. The family Rosaceae includes the highest number of genera that produce the compound, including *Cotoneaster* [50], *Eriobotrya* [53], *Fragaria* [57], *Malus* [77], *Prunophora* [67], *Pyrus* [103], *Rosa* [126] and *Sorbaria* [140] (Table 1).

The highest concentration (*ca.* 1.7%) of arbutin was found in the leaves of *Pyrus communis* [163]. Certain plants from families like Fabaceae [5,72,74,86], Lamiaceae [87,128,141] and Plantaginaceae [132,154,160] are also notable sources of this hydroquinone glycoside (Table 1). At least three genera of each of the families Rutaceae [48,94,161] and Saxifragaceae [22,36,131] are known to produce arbutin (Table 1). While leaves are the main source of the compound, it is present in other plant parts, e.g., aerial parts, flowers, fruits, stem and twigs (Table 1). The presence of arbutin in roots was only reported in *Picrorhiza scrophulariiflora* [96].

**Scheme 1:**
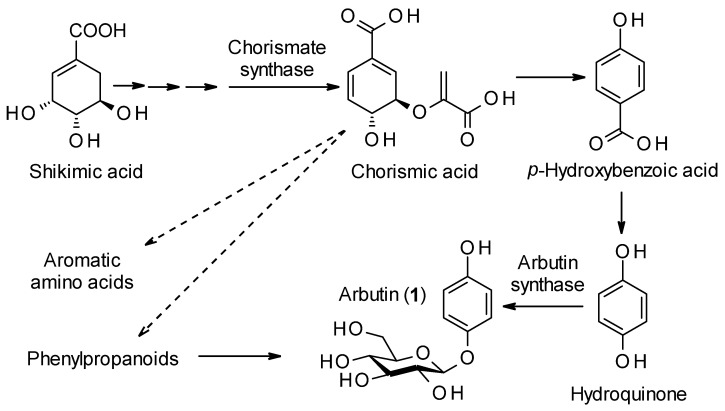
Biosynthesis of arbutin [164,165].

Grisdale and Towers [163] demonstrated that arbutin is biosynthesized in the young leaves of *Pyrus communis* and *Grevillea robusta* from shikimic acid, as well as from phenylpropanoid compounds (Scheme 1). Evidence has suggested that the hydroquinone skeleton could have been formed by the removal of the propyl side chain of certain phenylpropane derivatives, e.g., cinnamic acid and phenylalanine. However, there are several reports available in the literature that describe various engineered and artificial methods for enhanced biosynthesis of arbutin [164]. For example, Shen et al. [165] demonstrated an artificial pathway in *Escherichia coli* for increased production of arbutin from simple carbon sources.

## 3. Anticancer Potential of Arbutin

**Figure 2:**
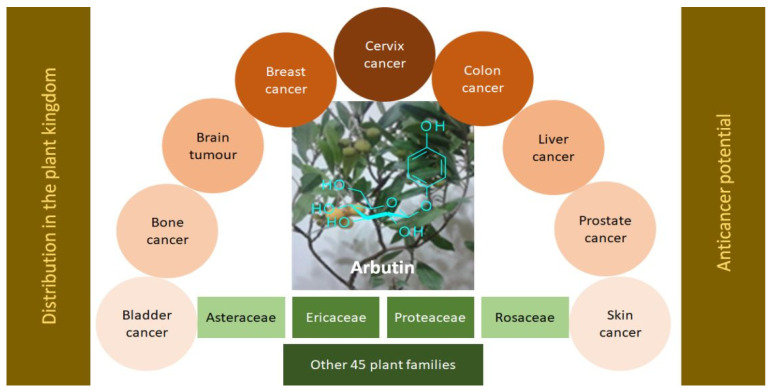
A schematic summary of the anticancer potential of arbutin, obtained from different plant families.

In addition to its skin whitening property which has been known for at least seven decades, arbutin (**1**) has been shown to possess various other therapeutically relevant biological properties, e.g., antioxidant, antimicrobial and anti-inflammatory [164,165]; it also has the potential as an anticancer agent [166,167,168,169,170,171,172,173,174,175,176,177,178,179,180,181]. Information obtained from the published literature on arbutin shows that this compound possesses cytotoxic properties against several human cancer and tumor cell lines including bladder, bone, brain, breast, cervical, colon, gastric, liver, prostate and skin cancers (Table 2) [166,167,168,169,170,171,172,173,174,175,176,177,178,179,180,181]. Most of these activities have been demonstrated in vitro, and in some cases, plausible mechanisms of action, e.g., apoptosis, have been identified (Table 2). A pictorial summary is presented in Figure 2. The activity of arbutin against various cancer cell lines is discussed in the following subsections.

### 3.1. Bladder Cancer

When malignant cells are formed in bladder tissue or lining, it is known as bladder cancer; this disease affects more than 10,000 people every year in the UK [182]. A study conducted with the TCCSUP (an anaplastic transitional cell carcinoma in the neck of the urinary bladder) human bladder cancer cell line revealed that arbutin did not have any cytotoxicity against this cell line at a concentration of <500 mg/mL, but it considerably decreased proliferation of this cell line in a concentration- and time-dependent manner in the MTT [3-(4,5-dimethylthiazol-2-yl)-2,5-diphenyltetrazolium bromide] assay [166]. It was also shown that arbutin could time-dependently disrupt the cell cycle and inactivate extracellular signal-regulated kinase (ERK), which is an intrinsic regulator of cell proliferation and a key mediator of p53-dependent cell cycle arrest [183]. The ERK signaling pathway is implicated in the mitogenic signaling of several growth factors [166]. It was postulated that the cell cycle disruption by arbutin could be mediated by an increase in the cyclin-dependent kinase inhibitor p21(WAF1/C1P1)(p21). That study demonstrated that arbutin could inhibit the cell proliferation of bladder cancer cells in vitro via extracellular signal-regulated kinase inactivation and p21 up-regulation [166,183].

### 3.2. Brain Tumour

In a recent study on the effect of arbutin on brain tumor, it was found that it could kill C6 glioma cells by inducing apoptosis (IC_50_ = 30 mM) and inhibiting the inflammatory markers and P13/AKT/mTOR cascade [167]. It should be noted that P13/AKT/mTOR is an intracellular signaling pathway that regulates the cell cycle and, thus, is linked to cell proliferation. It is known that reactive oxygen species (ROS) can activate this cascade [184]. It was demonstrated that arbutin-generated excessive ROS could disrupt the mitochondrial membrane, resulting in apoptosis in cells and inhibition of the cell adhesion property of C6 glioma cells. C6 glioma cells are spindle-like cells; they are able to stimulate human glioblastoma multiforme (GBM) when injected into the brain of neonatal rats and have been used to develop a glioma model in Wistar rats. These cells exhibit the same histological features as human GBM [185]. Like bladder cancer, over 11,000 people are diagnosed with primary brain tumors every year in the UK, and a half of those are cancerous [186]. A recent study [167] suggested that arbutin could be a potential anti-brain tumor drug for the treatment of glioma. However, further studies are obviously necessary in this regard. An earlier study also showed significant antiproliferative activity of arbutin against C6 rat brain tumor cells in an enzyme-linked immunosorbent assay (ELISA) [168].

### 3.3. Breast Cancer

Breast cancer is the most common type of cancer in the UK and is usually treated with chemotherapy and radiotherapy [187]. In the search for natural products as potential cures for breast cancer, the cytotoxicity of an arbutin-containing methanol extract of *Turnera diffusa* was evaluated using the MTT assay against epithelial-like MDA-MB-231 breast cancer cells; the IC_50_ value was determined to be 30.67 mg/mL [145]. It was also assessed against the human breast carcinoma T-47D cell line, showing an IC_50_ value of 54.02 mg/mL. It was demonstrated that the cytotoxic effect of an arbutin-containing extract was mediated via apoptosis. It is worth noting that T-47D are epithelial cells obtained from a pleural effusion from a 54-year-old female patient with an infiltrating ductal carcinoma of the breast [188]. This assessment did not use purified arbutin, but rather, tested a crude methanol extract that contained arbutin as well as the flavone apigenin. More recently, Hazman et al. [169] reported the cytotoxicity of purified arbutin against the MCF-7 human breast cancer cell line; cytotoxicity was shown to be mediated through the induction of apoptosis via estrogen receptors and the alpha signal pathway, as well as through inflammation and genotoxicity. It was observed that the administration of a lethal dose (LD_50_ = 69.6 mM) of 50% arbutin could induce inflammation in MCF-7 cells linked to pro-inflammatory cytokine levels and increase genotoxicity in the cells. It was noted, however, that while at high doses arbutin could induce apoptosis, at low concentrations, it had the opposite effect, i.e., inhibiting apoptosis and thus, assisting cancer cell growth and survival. Earlier, a similar study was conducted to determine the cytotoxicity of arbutin against adriamycin-resistant MCF-7 and wild-type MCF-7 cell lines using the MTT assay [170]. It was found that arbutin at a high concentration (5–10 mM) was the least cytotoxic (15–42% inhibition of cell growth) among the tested phenolic compounds against both cell lines, while at low concentrations (0.32–1.25 mM), this compound raised cell viability by approximately 20%. The effective concentrations (EC_50_) of arbutin against the adriamycin-resistant MCF-7 and wild-type MCF-7 cell lines were 5.85 mM and >1000 mM, respectively.

### 3.4. Cervical Cancer

Cervical cancer is cancer of the cervix, caused predominantly by infection from certain human papillomaviruses [189]. This cancer is most common among young females under 45 years of age. An arbutin-rich methanolic extract of the leaves of *Turnera diffusa*, i.e., not purified arbutin, was tested for its cytotoxicity against human cervical carcinoma HPV-16 positive (SiHa) and HPV negative (C-33) tumor cell lines. Its cytotoxicity against these cell lines was much less prominent than its effect against the MDA-MB-231 breast cancer cell line [145]. The IC_50_ values of this methanol extract against the SiHa and C-33 cell lines were 50.14 and 40.1 mg/mL, respectively. A year later, Erenler et al. [168] reported the antiproliferative property of purified arbutin against the HeLa cell line, which was first developed from cervical cancer cells in 1951. A real-time cell analyzer single plate instrument (RTCA) and electronic cell sensory array, the xCELLigence RTCA, were used to analyze this antiproliferative effect at concentrations of 10, 50 and 100 mg/mL against the HeLA cell line; however, no attempt was made to determine the IC_50_ value. Additionally, none of the above experiments explored the possible mechanism of action of arbutin against the human cervical cell lines.

### 3.5. Colon Cancer

Arbutin displayed cytotoxicity against HCT-15 cell line, a quasidiploid human cell line derived from the large intestine of a male colorectal cancer patient [171]. In that study, culture cells were incubated with various concentrations of this hydroquinone glycoside for four days in a 5% CO_2_ incubator before cell numbers were counted. However, since this preliminary cytotoxicity result [171], no follow up data on the cytotoxicity of arbutin against various other colon cancer cell lines have been published in the literature, despite the fact that colon cancer, also known as bowel cancer, is one of the most common types of cancer among people of over 60 years of age in the UK [190].

### 3.6. Gastric Cancer

Gastric cancer, a form of stomach cancer, is the disease in which cancer cells grow in the lining of the stomach, whereas stomach cancer can be found anywhere in the stomach. This form of cancer is not common in the UK [191]. The inhibitory effect of several derivatives of arbutin, isolated from the leaves of *Heliciopsis lobata*, against cultured gastric carcinoma MGC-803 cells invasion was reported by Qi et al. [63]. All these derivatives contained various acyl substituents on the glycone moiety of arbutin, e.g., cinnamoyl and butenyl. Most of these compounds displayed a moderate level of activity, with IC_50_ values between 11 and 45 mg/mL.

### 3.7. Liver Cancer

While most of the aforementioned potential anticancer activities were assessed in vitro, recently, Zeng et al. [172] reported in vivo anticancer activity of arbutin against diethylnitrosamine-initiated liver carcinogenesis in rats. Liver cancer is one of the leading causes of cancer deaths worldwide and is the sixth most common form of cancer in humans, with almost a million new cases in 2020 [172,192]. The administration (30 mg/kg body weight) of arbutin was found to improve body weight, reduce liver weight, improve the albumin, globulin and total protein contents, reduce liver injury marker enzyme function and increase the c-JNK (c-Jun *N*-terminal kinase), caspase-8 and p53 contents in rats with diethylnitrosamine-triggered liver carcinogenesis.

This effect was attributed to the anti-inflammatory and antioxidant properties of arbutin, as evident from a series of in vitro bioassays with isolated rat liver tissue involving various inflammatory markers. Furthermore, arbutin was shown to decrease the expression of GRP78 (78-kDa glucose-regulated protein), PDIA4 (protein disulfide isomerase family A member 4), GRP94 (94-kDa glucose-regulated protein), ERDJ4 (endoplasmic reticulum-localized DNA J4), ATF4 (activating transcription factor 4) and GADD34 (growth arrest and DNA damage-inducible protein 34) in liver tissues. Earlier, a similar in vivo experiment, albeit a preliminary one, was conducted with hydroquinone, which is the aglycone of arbutin [173]. It was reported that hydroquinone could inhibit the initiation of DNA-reactive carcinogen acetylaminofluorene induction of rat liver carcinogenesis. However, the authors did not observe any significant body weight gains or changes in liver weight in hydroquinone-treated rats.

In addition to the above in vivo studies, there are a few in vitro studies available in the literature where the effect of arbutin was studied against the HepG2 hepatocellular cancer cell line [145,174,175]. An arbutin-rich methanolic extract of the leaves of *Turnera difusa* was found to exert cytotoxicity toward the HepG2 cell line with an IC_50_ value of 43.87 mg/mL [145]. Hazman et al. [174] reported the effects of α-arbutin (but not β-arbutin) on HepG2 cells and cisplatin toxication in this cell line. At low doses, α-arbutin did not show any genotoxicity or cytotoxicity toward HepG2 cells, and no effects on apoptosis, inflammation or proliferation were observed. However, when the same low dose was used with cisplatin, oxidative stress, inflammation and genotoxicity levels increased, resulting from cisplatin toxicity without any change in caspase 3 levels. At high doses, α-arbutin displayed anticarcinogenic effects, mediated through increased oxidative stress, genotoxicity, inflammation and apoptosis and suppression of cell proliferation. A decade before this study, Kang et al. [175] reported on the in vitro cytotoxicity of arbutin in the HepG2 cell line.

### 3.8. Melanoma or Skin Cancer

Melanoma is a type of skin cancer, the most common sign of which is the appearance of a new mole or a noticeable change in an existing mole [193]. Melanoma is thought to be caused by exposure to ultraviolet (UV) light from the sun or from a sunbed. It is the fifth most common cancer in the UK and there are ca. 16,000 new cases of it reported in the UK every year. Jiang et al. [176] reported the potential anti-melanoma activity of arbutin and showed its effect on melanogenesis, as well as its pro-apoptotic effect, on B16 murine melanoma cells. Arbutin was shown to significantly reduce cell viability, promote cell apoptosis, cause G1 cell cycle arrest (after 24 h of treatment) and induce mitochondrial disruption in B16 murine melanoma cells. It also caused a reduction in the expression of B-cell lymphoma-extra large (Bcl-xL) and Bcl-2 arbutin-treated cells. The inhibition of cell viability by arbutin was found to be time- and dose-dependent, and it could inhibit melanogenesis by ca. 46% at a concentration of 5.4 mM. Its pro-apoptotic effect was detected by flow cytometry using Annexin V-FITC labeling for the detection of phosphatidylserine externalization. Arbutin was found to be able to cause apoptosis in 23.7% of the cells after 24 h of treatment at 5.4 mM. The results from this study indicated that arbutin could be a candidate for anti-melanoma drug development. Earlier, the anti-skin cancer potential of arbutin was reported by studying the molecular spectroscopic behavior of this compound and its action on the carcinogen *trans*-crotonaldehyde [177].

### 3.9. Osteosarcoma

Osteosarcoma is a type of bone cancer. It starts in the cells that form bones, especially long bones. Children, teens and young adults are the main sufferers from this cancer [194]. Just over 500 new cases are reported each year in the UK National Health Service (NHS) [195]. Wang et al. [178] demonstrated that arbutin could time- and dose-dependently suppress the progression of osteosarcoma in vitro using the osteosarcoma cell lines MG-63 and SW1353 and applying the Cell Counting Kit-8 assay. It was suggested that arbutin could inhibit osteosarcoma cell proliferation, migration and invasion via *miR-338-3pl* MTHFD1L (methylenetetrahydrofolate dehydrogenase (NADP+ Dependent) 1 Like) and by inactivating the AKT (protein kinase B)/mTOR (mammalian target of rapamycin) signaling pathway.

### 3.10. Prostate Cancer

Safari et al. [179] first reported the anti-prostate cancer potential of arbutin and looked into the molecular mechanism of activity against the prostate cancer cell line LNCap (androgen-sensitive human prostate adenocarcinoma cells). It was demonstrated that 1 mM of arbutin could induce apoptosis, reduce the level of reactive oxygen and decrease the expression of pro-inflammatory 1L-1β (interleukin-1 beta) and TNF-α (tumor necrosis factor alpha) genes. A year later, the effect of arbutin on the expression of tumor suppressor p53, BAX/BCL-2 (BCL 2 associated X/B cell lymphoma protein 2) ratio and oxidative stress induced by *t*-butyl hydroperoxide in fibroblast and LNCap cell lines was studied [180]. It was observed that arbutin could enhance the total antioxidant power and cell viability in the MTT assay, as well as reducing the BAX/BCL-2 ratio, p53 mRNA expression and necrosis in fibroblasts exposed to an oxidative agent. Additionally, it was shown to decrease cell viability, induce apoptosis and increase the BAX/BCL-2 ratio in LNCap cells at certain concentrations (e.g., 1 mM).

Recently, a dichloromethane extract of the leaves of *Arbutus pavarii* was shown to possess cytotoxicity against the PC3 human prostate cancer cell line. Employing a bioassay-guided isolation protocol, arbutin was isolated as one of the major bioactive compounds [12]. One in eight men in the UK is likely to have prostate cancer, which can develop when cells in the prostate start to grow in an uncontrolled way [196]. Prostate cancer is the most common cancer in men and more than 52,000 men are diagnosed with it every year in the UK. Fatalities from this disease every year in the UK are over 12,000. The in vitro activity of arbutin against prostate cancer cell lines requires further extensive investigation to examine the potential of this compound or its analogues as prostate cancer therapeutics.

### 3.11. Miscellaneous

In discussing the regulatory impact of miRNA-338-3p on cancer growth and migration, the antitumor effect of arbutin, i.e., suppressing cancer progression by promoting the expression of miRNA-338-3p, was highlighted by Mirzaei et al. [181].

## 4. Toxicological Aspects

Generally, arbutin is considered safe for external use, particularly at the concentrations at which it is used in various cosmetic products. However, a few studies conducted to date on the toxicity of this compound have reveled certain levels of in vivo and in vitro toxicity at various concentrations [197]. At high doses, the aglycone hydroquinone can exert hepato- and nephron-toxicity and mutagenicity [197]. Kang et al. [175] demonstrated the ability of arbutin to induce immunotoxicity in splenocyte cultures from mice. The genotoxic effect of arbutin on the differential gene expression profiling in A375 human malignant melanoma cells through its effect on tumorigenesis and related side-effects has been reported [198]. It was found that the level of toxicity may be dependent on the route of exposure, as well as on the sex, species and strain in rodents. Meanwhile, the subchronic and chronic toxicity in animal models was limited to nephrotoxicity [199]. However, no developmental and reproductive toxicity or carcinogenicity have been detected with arbutin [200,201]. Information available in various online databases suggests that it may exert a low level of toxicity at high doses when given orally to mice (LD_50_ = 9804 mg/kg) and rats (LD_50_ = 8715 mg/kg) [202], as well as dermal toxicity in rat and mouse (LD_50_ = 928 mg/kg). However, far more published papers have highlighted various protective and health promoting effects of arbutin, e.g., cytoprotective and hepatoprotective effects [103,202,203,204], the benefits of which probably outweigh the minimal toxic effect of this compound.

## 5. Conclusions

Arbutin is widely distributed in the plant kingdom; plants from the Asteraceae, Ericaceae, Proteaceae and Rosaceae families are the main sources of this compound. However, the compound has been detected in at least 45 other plant families to date. Published data suggest that arbutin possesses potential anticancer properties against bladder, bone, brain, breast, cervix, colon, liver, prostate and skin cancers, and a low level of toxicity. Further in silico studies and in vivo pre-clinical and randomized clinical investigations are essential to establish its true potential as an anticancer drug candidate.

## Figures and Tables

**Table 1:** Distribution of arbutin (**1**) in the plant kingdom.

Species	Family	Common Name	Plant Part	Geographical Source	Reference
*Aesculus californica* Nutt.	Hippocastanaceae	California buckeye	Fruit endosperm	USA	[4]
*Afgekia mahidolae* B.L. Burtt & Chermsir.	Fabaceae	Kan Pai Mahidol	Leaves	Thailand	[5]
*Ailanthus altissima* (Mill.) Swingle	Simaroubaceae	Varnish tree	Fruits	China	[6]
*Ainsliaea bonatii* Beauverd	Asteraceae	Chinese daisy	Leaves	China	[7]
*Amaranthus* spp.	Amaranthaceae	Amaranth	Leaves	Bangladesh	[8]
*Amaranthus tricolor* L.	Amaranthaceae	Amaranth	Leaves	Russia	[9]
*Antidesma thwaitesianum* Muell. Arg.	Phyllanthaceae	Mao tree	Fruits and leaves	Thailand	[10]
*Arbutus andrachne* L.	Ericaceae	Greek strawberry tree	Leaves	Greece and Turkey	[11]
*Arbutus pavarii* Pamp.	Ericaceae	Libyan strawberry tree	Leaves	Libya	[12]
*Arbutus unedo* L.	Ericaceae	Strawberry tree	Leaves	Mediterranean region and western Europe	[1,13]
			Fruits		[14]
*Arctostaphylos pungens* Kunth.	Ericaceae	Point leaf manzanita	Leaves	Italy, Mexico and USA	[15]
*Arctostaphylos* spp.	Ericaceae	Bearberry	Leaves	Scotland and Scandinavia	[16,17]
*Arctostaphylos uva-ursi* (L.) Spreng.	Ericaceae	Bearberry	Leaves	Bulgaria, Turkey	[18]
*Arctous alpina* (L.) Nied.	Ericaceae	Alpine bearberry	Leaves	Russia	[19]
*Artemisia pallens* Wall. Ex. DC.	Asteraceae	Damanaka	Leaves	India	[20]
*Artocarpus lacucha* L.	Moraceae	Monkey fruit	Leaves	South-east Asia	[21]
*Astilbe rivularis* L.	Saxifragaceae	False spirea	Leaves	Nepal and UK	[22]
*Atriplex littoralis* L.	Amaranthaceae	Grass leaf orache	Aerial parts	Serbia	[23]
*Bacopa procumbens* (Mill.) Greenm.	Plantaginaceae	Baby jump-up	Aerial parts	Tropical and subtropical areas of North and South America	[24]
*Bellendena montana* R. Br.	Proteaceae	Mountain rocket	Leaves	Tasmania	[25]
*Benincasa hispida* (Thunb.) Cogn.	Cucurbitaceae	Wax gourd	Fruits	China	[26]
*Bergenia ciliata* (Haw.) Sternb.	Saxifragaceae	Fringed elephant’s ears	Rhizome	Nepal	[27]
*Bergenia cordifolia* L.	Saxifragaceae	Heartleaf Bergenia	Leaves	Russia	[28]
*Bergenia crassifolia* (L.) Fritsch.	Saxifragaceae	Heart-leaved Bergenia	Aerial parts	Russia	[29]
			Leaves	Russia	[30]
			Leaves	Romania	[31]
*Bergenia purpurascens* (Hook. f. & Thomson) Engl.	Saxifragaceae	Purple Bergenia	Leaves	China	[32]
*Bergenia* spp.	Saxifragaceae	Elephant’s ears	Aerial parts	Afghanistan to China and the Himalayan region	[17,33,34,35]
*Bergenia stracheyi* (Hook. F. & Thoms.) Engl.	Saxifragaceae	Elephant’s ears	Aerial parts	The Himalayas	[36]
*Betula pendula* Roth.	Betulaceae	Silver birch	Leaves	Europe and Asia	[37]
*Betula platyphylla* Sukatchev var. *japonica* Hara	Betulaceae	Shirakamba	Leaves	China	[38]
*Betula schmidtii* Regel.	Betulaceae	Schmidt’s birch	Bark	China, Japan, Korea and Russia	[39]
*Breynia officinalis* Hemsl.	Phyllanthaceae	Chi R Yun	Leaves	China and Japan	[40]
*Breynia rostrata* Merr.	Phyllanthaceae	Hui Guo Hei Mian Shen	Aerial parts	China and Vietnam	[41]
*Calluna* spp.	Ericaceae	Heather	Leaves	Europe and Asia Minor	[17]
*Calluna vulgaris* L. Hull.	Ericaceae	Heather	Aerial parts	Asia Minor	[42]
			Leaves	Russia	[43]
*Careya arborea* Roxb.	Lecythidaceae	Slow match tree	Bark, leaves and seeds	India	[44]
*Casearia multinervosa* C.T.White & Sleumer	Salicaceae	Casearia	Stem	Australia	[45]
*Cenarrhenes nitida* R. Br.	Proteaceae	Port Arthur plum	Leaves	Tasmania	[25]
*Centaurea urvillei* DC. subsp. *urvillei*	Asteraceae	Star thistle	Leaves	Turkey	[46]
*Chamaecyparis lawsoniana*	Cupressaceae	Lawson cypress	Galls	Iran	[47]
*Clausena indica* (Datz.) Oliver	Rutaceae	Indian wampi	Fruit pericarp	India and Sri Lanka	[48]
*Coriandrum sativum* L.	Apiaceae	Coriander	Aerial parts	Western Asia, Southern Europe and Russia	[49]
*Cotoneaster simonsii* Baker	Rosaceae	Himalayan cotoneaster	Aerial parts	The Himalayas	[50]
*Cuscuta sinensis* Lam.	Convolvulaceae	Chinese cuscuta	Semen	China, Japan and Korea	[51]
*Dryopteris sublaeta* Ching & Y. P. Hsu	Dryopteridaceae	Chinese male fern	Rhizome	China	[52]
*Eriobotrya fragrans* Champ. Ex. Benth.	Rosaceae	Xiang hua pi ba	Leaves	China and Vietnam	[53]
*Eryngium bourgatii* Gouan.	Apiaceae	Sea holly	Flowers and leaves	Spain	[54]
*Eugenia hyemalis* L. Cambess	Myrtaceae	Hyemalis	Aerial parts	Argentina, Bolivia and USA	[55]
*Flammulina velutipes* (Curtis) Singer	Physalacriaceae	Velvet shank	Leaves	China	[56]
*Fragaria* spp.	Rosaceae	Strawberry	Roots	Europe, North America and China	[57]
*Gentiana pyrenaica* L.	Gentianaceae	Pyrenian gentian	Leaves	United Kingdom	[58]
*Grevillea banksii* R. Br.	Proteaceae	Dwarf silky oak	Leaves	Australia	[59]
*Grevillea robusta* A. Cunn. Ex R. Br.	Proteaceae	Silk oak	Leaves	Australia and India	[60]
			Bark and leaves		[61]
*Hakea saligna* L.	Proteaceae	Hakea	Leaves	Australia and India	[60]
*Halocarpus biformis* (Hook.) C.J. Quinn	Podocarpaceae	Yellow pine	Leaves	New Zealand	[62]
*Heliciopsis lobata* (Merr.) Sleumer	Proteaceae	Helicia	Leaves	China and Vietnam	[63]
*Herpetospermum caudigerum* Wall.	Cucurbitaceae	Herpetospermum	Leaves	China, India and Tibet	[64]
*Homalium zeylanicum* (Gardner) Benth.	Flacourtiaceae	Kalavaram	Leaves	India	[65]
*Huperzia serrata*	Lycopodiaceae	Toothed clubmoss	Whole plant	China, Japan, Korea, Russia and Tibet	[66]
*Ilex brasiliensis* (Spreng.) Loes.	Aquifoliaceae	Brazilian holly	Leaves	Brazil	[67]
*Ilex integerrima* Reiss.	Aquifoliaceae	Holly	Leaves	Brazil	[67]
*Ilex latifolia* Thunb.	Aquifoliaceae	Tarajo holly	Leaves	Japan	[68]
*Ilex pseudobuxus* Reiss.	Aquifoliaceae	Brazilian holly	Leaves	Brazil	[67]
*Ilex theezans* Mart.	Aquifoliaceae	Congonha	Leaves	Brazil	[67]
*Jamesia americana* Torr. & A. Gray	Hydrangeaceae	Cliffbush	Aerial parts	USA	[69]
*Juglans regia* L.	Juglandaceae	Walnuts	Nuts	The Balkans, the Himalayans and China	[70]
*Larix leptolepis*	Pinaceae	Japanese Larch	Needles	Japan	[71]
*Lens culinaris* Medik.	Fabaceae	Lentil	Seeds	India	[72]
*Leucadendron* spp.	Proteaceae	Conebushes	Leaves	South Africa	[73]
*Lysiloma latisiliquum* (L.) Benth.	Fabaceae	Wild tamarind	Leaves	USA	[74]
*Madhuca latifolia* (J. Konig) J.F. Macbr.	Sapotaceae	Mahua	Seeds	India, Nepal, Pakistan and Sri Lanka	[75]
*Magnifera indica* L.	Anacardiaceae	Mango	Leaves	India	[76]
*Malus sylvestris* (L.) Mill.	Rosaceae	Crab apple	Leaves	United Kingdom & Russia	[77]
		Crab apple	Fruits	Russia	[78]
*Morus alba* L.	Moraceae	Mulberry	Leaves	China and India	[79]
*Mutisia acuminata* var. *acuminata* Ruiz & Pav.	Asteraceae	Bolivian Mutisia	Aerial parts	Peru and Bolivia	[80]
*Mutisia acuminata* var. *hirsuta* (Meyen) Cabrera	Asteraceae	Mutisia	Leaves	Peru	[81]
*Myrsine seguinii* H. Lev.	Myrsinaceae *alt.* Primulaceae	Myrsine	Leaves	China, Japan and New Zealand	[82]
*Myrothamnus flabellifolia* Welw.	Myrothamnaceae	Resurrection plant	Leaves	South Africa	[83]
			Aerial parts	Germany	[84]
*Onobrychis kachetica* Boiss. & Buhse	Fabaceae	Espartzet Kakhetinski	Leaves	Trans-caucasus, and Russia	[85]
*Onobrychis viciifolia* Scop.	Fabaceae	Sainfoin	Petals	Euro Siberian temperate region	[86]
*Origanum dubium* Boiss.	Lamiaceae	Rouvanos	Aerial parts	Cyprus	[87]
*Origanum majorana* L.	Lamiaceae	Sweet majoram	Leaves	Egypt	[88]
*Origanum vulgare* L.	Lamiaceae	Oregano or wild majoram	Aerial parts	Mediterranean region	[89]
*Paederia scandens* (Loir.) Merr.	Rubiaceae	Gandheli	Aerial parts	China and India	[90]
*Paulownia fortune* (Seem.) Hemsl.	Paulowniaceae	Dragon tree	Flowers	China	[91]
*Persoonia gunnii* Hook. f.	Proteaceae	Persoonia	Leaves	Tasmania	[25]
*Petasites tricholobus* Franch.	Asteraceae	Butterburs	Aerial parts	China, Nepal, Pakistan and Vietnam	[92]
*Phellinus linteus* (Berk. & M.A. Curtis) Teng	Hymenochaetaceae	Meshimakobu	Aerial parts	China, Korea and Japan	[93]
*Phellodendron chinense* var. *glabriusculum* C.K. Schenid.	Rutaceae	Cork tree	Aerial parts	China	[94]
*Phyllostachys heterocycla* Mitf.	Poaceae	Mousouchiku or tortoise shell bamboo	Bamboo-sheath	Japan	[95]
*Picrorhiza scrophulariiflora* Pennell.	Scrophulariaceae	Xizang Huhuanglian	Roots	China, India and Tibet	[96]
*Platycodon grandiflorum* L.	Campanulaceae	Balloon flower	Leaves	China	[97]
*Podospermum canum* C. A. Mey	Asteraceae	Karakok	Aerial parts	Caucasia, Iran, Iraq, Syria and Turkey	[98]
*Prunophora salicina* Linn.	Rosaceae	Chinese Plum	Fruit peels	China and Korea	[67]
*Psophocarpus tetragonolobus* (L.) DC	Fabaceae	Winged bean	Leaves	India	[99]
*Pyrola calliantha* Andres	Ericaceae	Wintergreen	Leaves	Eastern Himalaya to China	[100]
*Pyrola incarnata* Fisch.	Ericaceae	*Lu Shou Cha*	Leaves	China	[101]
*Pyrus anatolica* Browicz	Rosaceae	Turkish pear	Fruits, leaves and stem	Turkey	[102]
*Pyrus biossieriana* Buhse	Rosaceae	Wild pear	Leaves	Iran	[103]
*Pyrus bretschneideri* Rehder	Rosaceae	Ya pear	Leaves	China	[104]
*Pyrus bourgaeana* Decne.	Rosaceae	Iberian pear	Aerial parts	Iberian Peninsula and Morocco	[105]
*Pyrus communis* L.	Rosaceae	Pear or Rocha pear	Leaves	Central and eastern Europe and western Asia	[106,107]
			Aerial parts and seeds		[108]
			Flowers	Poland	[109]
*Pyrus communis* L. var. *sativa* (DC.)	Rosaceae	Pear	Twigs	China	[110]
*Pyrus communis* L. *cv. Wujiuxiang*	Rosaceae	Wujiuxiang pear	Fruit peels	China	[111]
*Pyrus elaeagrifolia* Pall.	Rosaceae	Wild pear	Leaves	Albania, Bulgaria, Romania and Turkey	[112]
*Pyrus pashia* Buch ham ex D. Don	Rosaceae	Kainth	Fruits	The Himalayas	[113]
*Pyrus pyraster* (L.) Burgsd.	Rosaceae	European wild pear	Fruit peels	Western Europe to the Caucasus	[114,115]
*Pyrus pyrifolia* Nakai	Rosaceae	Niitaka or Asian pear	Fruits	Japan	[104,116]
			Fruits	Korea	[117]
		Asian pear	Fruit peels	China	[118]
*Pyrus pyrifolia* cv. *Kousui* Nakai	Rosaceae	Japanese pear	Branches, fruits, leaves and stem	Japan	[119]
*Pyrus serotina* Rehder. var. *culta* Rehdar.	Rosaceae	Japanese pear	Leaves	Japan	[120]
*Pyrus spinosa*	Rosaceae	Almond-leaved pear	Twigs	Siberia	[115]
*Pyrus* spp.	Rosaceae	Pear	Stem	Central and eastern Europe and western Asia	[121]
*Pyrus ussuriensis* Maxim.	Rosaceae	Ussurian pear	Leaves	China	[104]
*Rhodiola coccinea* (Royle) Boriss.	Crassulaceae	Rhodiola	Aerial parts	Central Asia, south-western Siberia and central China	[122]
*Rhodiola crenulata* LLL	Crassulaceae	Arctic root	Aerial parts	China	[123]
*Rhodiola rosea* L.	Crassulaceae	Golden root	Aerial parts	China	[124]
*Rhododendron adamsii* Rehder	Ericaceae	Sagaan dali	Leaves	Russia	[125]
*Rhododendron dauricum* L.	Ericaceae	Dauria	Leaves	China, Mongolia and Russia	[125]
*Rhododendron fauriei* Franch. *var. brachycarpum*	Ericaceae	Japanese Rhododendron	Leaves	Japan, Korea and Russia	[125]
*Rhododendron luteum* Sweet	Ericaceae	Yellow azalea	Leaves	Poland and Russia	[125]
*Rhododendron ponticum* L.	Ericaceae	Common rhododendron	Leaves	Iberian Peninsula and Russia	[125]
*Rosa roxburghii* Tratt.	Rosaceae	Roxburgh rose	Leaves	China	[126]
*Salix acmophylla* Boiss.	Salicaceae	Brook willow	Aerial parts	Pakistan and central Asia	[127]
*Salvia hispanica* L.	Lamiaceae	Chia	Flowers and stem	Central America	[128]
*Salvia mexicana* var. *Mexicana* L.	Lamiaceae	Mexican sage	Aerial parts	Mexico	[129]
*Sambucus nigra* L.	Adoxaceae	Elderberry or black elder	Fruits	Serbia	[130]
*Saxifraga stolonifera* Curtis	Saxifragaceae	Creeping sailor	Leaves	China, Japan and Korea	[131]
*Scrofella chinensis* Maxim.	Plantaginaceae	Scrofella	Whole plant	China	[132]
*Sedum purpureum* L.	Crassulaceae	Purple spoon-leaved stonecrop	Leaves	United Kingdom	[133]
*Sedum* spp.	Crassulaceae	Stonecrops	Leaves	Northern hemisphere	[134]
*Selaginella tamariscina* (Beauv.) Spring	Selaginellaceae	Selaginella	Aerial parts	China, India, Japan, Korea, Russia and Thailand	[135]
*Serratula komaroviilljin* L.	Asteraceae	Saw-wort	Leaves	Russia	[136]
*Serratula quinquefolia* M. Bieb. ex. Willd.	Asteraceae	Five-leaved saw-wort	Leaves	Poland	[137]
*Serratula sogdiana* (Bunge) L. Martins	Asteraceae	Plumeless saw-wort	Leaves	Eurasia	[138]
*Sonneratia alba* Sm.	Lythraceae	Perepat	Leaves	East Africa and south-east/far east Asia	[139]
*Sorbaria arborea* Schneid.	Rosaceae	False spirea	Stem	China	[140]
*Stachys alopecuros* (L.) Benth. Subsp. *divulsa* (Ten.) Grande	Lamiaceae	Yellow betony	Aerial parts	Italy	[141]
*Stachys germanica* L subsp. *Salviifolia* (Ten.) Gams.	Lamiaceae	Downy woundwort	Aerial parts	Italy and Germany	[142]
*Stachys lavandulifolia* Vahl.	Lamiaceae	Wood betony	Aerial parts	Iran	[143]
*Teucrium chamaedrys* L.	Lamiaceae	Wall germander	Leaves	Mediterranean region	[144]
*Turnera diffusa* Willd.	Passifloraceae	Damiana	Leaves and stem	Mexico and USA	[145]
*Vaccinium arctostaphylos* L.	Ericaceae	Caucasian whortleberry	Leaves	Armenia, Azerbaijan, Bulgaria, Georgia, Iran, Russia and Turkey	[146]
*Vaccinium dunalianum* Wight	Ericaceae	Chinese blueberry	Flower buds, fruits and leaves	Assam, China South-Central, China Southeast, East Himalaya, Myanmar, Nepal, Taiwan, Tibet and Vietnam	[147]
*Vaccinium myrtillus* L.	Ericaceae	European blueberry	Leaves and fruits	Europe	[148]
			Leaves and stem	Europe	[149]
*Vaccinium vacillans* Torr.	Ericaceae	Blueberry	Leaves	Rhode Island	[150]
*Vaccinium vitis-idaea* L.	Ericaceae	Cowberry	Leaves and berries	Alaska, Canada, Poland, Russia and Eurasia	[151,152]
			Aerial parts	China	[153]
*Veronica austriaca* L.	Plantaginaceae	Broadleaf speedwell	Leaves	Bulgaria	[154]
*Veronica turrilliana* Stoj. & Stef.	Plantaginaceae	Speedwell	Aerial parts	Bulgaria	[155]
*Viburnum fordiae* Hance	Viburnaceae	Bright red berry	Stem	China	[156]
*Viburnum opulus* L.	Viburnaceae	Guelder rose	Leaves	Europe, northern Africa and central Asia	[68,157]
*Viburnum phlebotrichum* Siebold & Zucc.	Viburnaceae	Japanese viburnum	Leaves	Japan	[68,158]
*Viola arvensis* L.	Violaceae	Field Pansy	Aerial parts	Russia	[159]
*Wulfeniopsis amherstiana* (Benth.) D.Y. Hong	Plantaginaceae	Himalyan Wulfenia	Leaves	The Himalayas	[160]
*Xanthoxylum piperitum* DC	Rutaceae	Sichuan pepper or Japanese pepper	Pericarp and seeds	Japan	[161]
*Zanthoxylum bungeanum* Maxim.	Rutaceae	Japanese pepper tree	Pericarps	China and Japan	[162]

**Table 2:** Cytotoxicity of arbutin (**1**) against various cancer and tumor cell lines.

Type of Cancer/Tumour	Brief Description of Anticancer Activity of Arbutin (1)	In Vivo/In Vitro	References
Bladder cancer	Inhibition of TCCSUP (anaplastic transitional cell carcinoma in the neck of the urinary bladder) bladder cancer cell proliferation.	In vitro	[166]
Brain tumour	Activity against rat C6 glioma cells.	In vitro	[167,168]
Breast cancer	Cytotoxicity of arbutin containing methanolic extract against MDA-MB-231 and T-47D breast cancer cells.	In vitro	[145]
	Cytotoxicity towards the MCF-7 (breast cancer) cell line.	In vitro	[169]
	Cytotoxicity against adriamycin-resistant MCF-7 and wild-type MCF-7.	In vitro	[170]
Cervical cancer	Antiproliferative activity against HeLa cells.	In vitro	[168]
	Activity against human cervical carcinoma HPV-16 positive (SiHa) and HPV negative (C-33) cell lines.		[145]
Colon cancer	Assessed for cytotoxicity against HCT-15 cells derived from human colon carcinoma.	In vitro	[171]
Gastric cancer	Inhibition of gastric carcinoma MGC-803 cells invasion.	In vitro	[63]
Liver cancer	Antioxidant, anti-inflammatory and anticancer activities against diethylnitrosamine-induced liver carcinoma in rats.	In vivo	[172]
	Inhibition of DNA-reactive carcinogen acetylaminofluorene induction of initiation of rat liver carcinogenesis.	In vivo	[173]
	Anticarcinogenic activity against hepatocellular carcinoma cells (HepG2).	In vitro	[169,174]
	Cytotoxicity against HepG2 cells.	In vitro	[175]
Skin cancer	Pro-apoptotic activity on B16 murine melanoma cells.	In vitro	[176]
	Action on the toxic *trans*-crotonaldehyde.	In vitro	[177]
Osteosarcoma	Suppression of osteosarcoma progression.	In vitro	[178]
Prostate cancer	Induction of apoptosis in human prostrate adenocarcinoma (LNCaP) cells.	In vitro	[179,180]
	Cytotoxicity against the prostate cancer cell line PC3.		[12]
Miscellaneous	Promotion of expression of miRNA-338-3p in suppressing cancer progression.	In vitro	[181]
